# Machine learning for classifying chronic kidney disease and predicting creatinine levels using at-home measurements

**DOI:** 10.1038/s41598-025-88631-y

**Published:** 2025-02-05

**Authors:** Brady Metherall, Anna K. Berryman, Georgia S. Brennan

**Affiliations:** Mathematical Institute, University of Oxford, Radcliffe Observatory Quarter, Andrew Wiles Building, Woodstock Rd, Oxford, OX2 6GG UK

**Keywords:** Chronic kidney disease classification, Creatinine prediction, Machine learning, At-home detection, Kidney diseases, Applied mathematics, Scientific data

## Abstract

Chronic kidney disease (CKD) is a global health concern with early detection playing a pivotal role in effective management. Machine learning models demonstrate promise in CKD detection, yet the impact on detection and classification using different sets of clinical features remains under-explored. In this study, we focus on CKD classification and creatinine prediction using three sets of features: at-home, monitoring, and laboratory. We employ artificial neural networks (ANNs) and random forests (RFs) on a dataset of 400 patients with 25 input features, which we divide into three feature sets. Using 10-fold cross-validation, we calculate metrics such as accuracy, true positive rate (TPR), true negative rate (TNR), and mean squared error. Our results reveal RF achieves superior accuracy (92.5%) in at-home CKD classification over ANNs (82.9%). ANNs achieve a higher TPR (92.0%), but a lower TNR (67.9%) compared with RFs (90.0% and 95.8%, respectively). For monitoring and laboratory features, both methods achieve accuracies exceeding 98%. The R2 score for creatinine regression is approximately 0.3 higher with laboratory features than at-home features. Feature importance analysis identifies the key clinical variables hemoglobin and blood urea, and key comorbidities hypertension and diabetes mellitus, in agreement with previous studies. Machine learning models, particularly RFs, exhibit promise in CKD diagnosis and highlight significant features in CKD detection. Moreover, such models may assist in screening a general population using at-home features—potentially increasing early detection of CKD, thus improving patient care and offering hope for a more effective approach to managing this prevalent health condition.

## Introduction

Chronic kidney disease (CKD) represents a global health challenge affecting millions worldwide and placing a substantial burden on healthcare systems^1,2^. More women are affected by CKD than breast cancer, and more men than prostate cancer^3^. CKD is often a silent and progressive condition remaining undetected until a significant loss of kidney function has occurred. Early detection and prediction are crucial for timely interventions and improved patient outcomes.

CKD is classified into five stages^4–6^ based on glomerular filtration rate (GFR)—a measure of kidney function. GFR measurement is complex and so is usually estimated using equations. The two most common equations are the Modification of Diet in Renal Disease (MDRD)^7^ and Chronic Kidney Disease Epidemiology Collaboration (CKD-EPI)^8^ equations. The MDRD equation has limitations for healthy individuals or those with mild kidney dysfunction. In contrast, the CKD-EPI equation addresses some of these limitations, providing a more accurate estimate of GFR by adjusting for different creatinine ranges.

Machine learning, which has shown success in predicting diseases^9^, holds promise within nephrology^10^, including enhancing CKD screening and detection^3,11–13^. One promising application of machine learning in the context of CKD is the potential for at-home detection or screening. Online CKD detection, such as through a health application on smartphones, is identified as an area for future research by Qezelbash-Cham et al.^3^. At-home CKD screening offers an ideal solution to the global health challenge posed by CKD. By leveraging user-friendly devices and predictive models, individuals could track key indicators of kidney health in the comfort of their homes. This approach not only increases early detection, but also enables a wider subset of the population to be screened. The convenience of at-home screening, coupled with the predictive capabilities of machine learning, could significantly improve the efficiency of CKD management, leading to better patient outcomes and alleviating the burden on healthcare systems worldwide. Moreover, such an approach aligns with the broader trend of personalized and preventive healthcare. However, such an application would be limited to more accessible features than in a clinical setting^11^.

Previous studies have primarily relied on either using all available clinical features or selecting a reduced set of features based on their statistical importance to train models effectively. However, in the context of at-home or online CKD detection, this approach faces significant limitations. Unlike a clinical setting, where a wide range of precise biomarkers and diagnostic tools are readily accessible, at-home applications are constrained to features that can be measured non-invasively and with minimal equipment. This restriction necessitates a re-evaluation of feature selection, prioritizing those that can be easily monitored by individuals at home, such as blood pressure, and potentially supplemented by data from regular checkups or wearable devices. To address these constraints, we categorize features not by their relative statistical importance, but by their accessibility and ease of measurement. This approach shifts the focus from optimal feature selection based on predictive value to a balanced model that leverages practical, accessible data while still providing accurate insights into CKD risk. Such a division of features by accessibility could help tailor machine learning models specifically for home-based applications, maximizing their relevance and usability for everyday users.

Our primary focus is detection of CKD with at-home measurements to facilitate easier and earlier detection throughout the general population. To the best of our knowledge there is no study comparing the performance of machine learning algorithms using the full feature set from laboratory tests and only the features that can be measured at home. To gain insight into the possible utility of machine learning for at-home detection we develop machine learning models for two purposes. First, we aim to classify whether a patient has CKD (by grouping all five stages together), and second, to predict creatinine levels, as is done in Ref.^14^, which can then be used to calculate the estimated GFR and consequently determine the stage of CKD. In this study, we categorize the features into three sets: at-home features (tests that can be easily conducted at home), monitoring features (encompassing basic testing), and laboratory features (including comprehensive tests). To achieve these goals we employ artificial neural networks and random forest. Artificial neural networks and random forest are two of the most commonly investigated and successful machine learning algorithms in the context of chronic kidney disease according to the review in Ref.^12^.

**Table 1:** Feature list organized into at-home, monitoring, and laboratory groups, where the at-home group is a subgroup of monitoring, and monitoring is a subgroup of laboratory features.

Attribute	Symbol	Type	Feature set
Age	Age	Numerical	At-home
Race	Race	Nominal	At-home
Sex	Sex	Nominal	At-home
Hypertension	HTN	Nominal	At-home
Diabetes mellitus	DM	Nominal	At-home
Coronary artery disease	CAD	Nominal	At-home
Appetite	APPET	Nominal	At-home
Pedal edema	PE	Nominal	At-home
Anemia	ANE	Nominal	At-home
Blood pressure	BP	Numerical	At-home
Red blood cells	RBC	Nominal	Monitoring
Red blood cell count	RBCC	Numerical	Monitoring
White blood cell count	WBCC	Numerical	Monitoring
Blood glucose random	BGR	Numerical	Monitoring
Blood urea	BU	Numerical	Monitoring
Sodium	SOD	Numerical	Monitoring
Potassium	POT	Numerical	Monitoring
Hemoglobin	HEMO	Numerical	Monitoring
Specific gravity	SG	Nominal	Laboratory
Albumin	AL	Nominal	Laboratory
Sugar	SU	Nominal	Laboratory
Bacteria	BA	Nominal	Laboratory
Pus cell	PC	Nominal	Laboratory
Pus cell clumps	PCC	Nominal	Laboratory
Packed cell volume	PCV	Numerical	Laboratory

## Methods

### Data

We use a publicly available dataset hosted on the University of California, Irvine (UCI) machine learning repository^15^. The dataset consists of 400 patients from a hospital in Tamil Nadu, India admitted over a period of two months. The dataset has been widely used to apply machine learning techniques to CKD detection and classification; over 50% of the studies in the review in Ref.^3^ have used this dataset. The data on each patient includes blood test results, in addition to comorbidity and demographic data. The dataset has 250 patients labelled as having CKD and 150 patients labelled without CKD. The labels were assigned by nephrologists based on patient history, symptoms, and blood and urine tests^16^. The dataset also includes serum creatinine, which is the key quantity in both the MDRD and CKD-EPI equations for estimated GFR. Furthermore, we supplement the data with sex and race data from Ref.^17^. We will use both the CKD indicator and serum creatinine measurement as dependent variables in our analysis. For descriptive statistics of the data see the Supplementary Information.

#### Feature sets

To help facilitate at-home diagnoses we separate the features into three sets as shown in Table 1. The smallest set is made up of features measurable at home (at-home features). At-home features include all patient demographic and comorbidity data, as well as blood pressure, which can be easily measured either at home or at a local pharmacy. The second set, monitoring features, are typically obtained if a patient is monitoring their health with health checks in a clinic. The monitoring set includes the at-home features as well as features measurable with standard tests (i.e. blood urea and blood glucose). Finally, the third set, which we call laboratory features, includes all 25 features, where the remaining features are measured with more specialized tests.

#### Pre-processing

Our pre-processing of the data involves three steps. First, we discard the 17 patients that do not have a serum creatinine reading since we cannot use these patients to train or test our regression models. Second, we impute the missing values of numerical features. Qin et al.^18^ investigated the impact of missing data imputation techniques on the prediction of CKD. They suggest using *k*-nearest neighbours (*k*-NN) imputation instead of mean imputation because of potential skewness in the data. The *k*-nearest neighbours algorithm finds the *k*-nearest neighbours in the space of complete features. The mean of these *k* neighbours is then used to impute the missing data. We use *k*-NN imputing with $k=5$ to deal with missing data in numerical features. Finally, we use one hot encoding to represent nominal features. One hot encoding takes nominal features with *n* classes and replaces the original feature column in the feature matrix with *n* columns each representing a single class. Each row contains one 1 in the column that corresponds to a patient’s class, and 0s in all the remaining columns. We treat any missing values in the nominal features as their own class in the one hot encoding instead of imputing their value.

We standardize the numerical features to have mean 0 and standard deviation 1 so the distribution of values is comparable across features. Furthermore, we use the log of serum creatinine for the regression. This ensures we predict only positive values for serum creatinine and gives us more uniformly distributed data.

### Machine learning algorithms

To evaluate the potential for at-home, early detection of CKD we undertake two investigations: first, focusing on the classification of CKD, and second, directly predicting creatinine levels. We investigate these two tasks on each feature set described in Table 1. To address these challenges, we employ two machine learning techniques: artificial neural networks (ANNs) and random forests (RFs).

ANNs are promising tools in clinical medicine and are inspired by biological neural networks. They consist of interconnected nodes organized into layers to process data and learn patterns. ANNs excel at recognizing complex patterns, making accurate predictions, and adapting to change. ANNs’ architecture includes input, hidden, and output layers, with weighted connections that are adjusted during training. However, challenges exist; ANNs often require large amounts of high-quality, diverse training data for optimal performance and generalization. They can lack interpretability, acting as black boxes, making decision processes unclear.

Alongside ANNs, we will test RF algorithms, an ensemble method combining multiple decision trees trained on subsets of the data and features. During prediction, RFs aggregate tree predictions using majority voting or averaging, thus reducing overfitting and enhancing generalization. RFs generally provide better interpretability than ANNs. The decision trees in RFs can be visualized, revealing learned rules and conditions, and measures of feature importance indicate each feature’s contribution.

### Model evaluation

We now introduce the loss functions we employ to optimize our models with respect to, as well as the metrics we will use to assess our model’s ability to classify CKD and predict creatinine levels. For binary classification, we use the loss function of cross-entropy, or log loss. Within an ANN, entropy gauges the disparity between the predicted probability of a binary outcome and the actual binary label, and for RF entropy determines the splitting criteria within a decision tree. In our evaluation of classification models on the test data we will employ five essential metrics. The primary metric is accuracy, which provides a basic measure of overall classification correctness, but may fall short in imbalanced class scenarios. Additionally, we use the true positive rate (TPR) to gauge the model’s effectiveness in correctly identifying positive cases and the true negative rate (TNR) to assess its proficiency in recognizing negative cases. We also consider the false positive rate (FPR) to measure the proportion of incorrect positive classifications. Finally, the false negative rate (FNR) to evaluate the model’s ability to avoid missing positive cases, especially where false negatives could have significant consequences, such as our application. These metrics collectively offer a comprehensive evaluation of a classification model’s performance. Receiver operating characteristics (ROC) curves are commonly used in medical decision making and increasingly in machine learning since simple accuracy is often a poor metric^19^. Typical machine learning classification algorithms yield a probability of a sample being in any class. Thus, the discrete class assigned to each sample depends on the threshold used for assigning a discrete class from the probability. ROC curves plot the true positive rate against the false positive rate as the threshold varies. ROC curves allow us to understand the ability of a classifier to rank positive instances above negative instances^19^. We follow the suggestion in Ref.^20^ and use vertical averaging to find the mean ROC curve across folds. It is common to reduce a ROC curve down to a single number—the area under the curve (AUC). The AUC is always between 0 and 1, where 1 is perfect performance, 0.5 is random guessing, and 0 *always mis-classifies* (which can be inverted to yield perfect performance).

To evaluate the performance of our creatinine regression models we employ three commonly used metrics. Mean squared error (MSE) measures the average squared difference between predicted and true values and has a sensitivity to larger errors and outliers. The R-squared (R2) score indicates the proportion of variance explained by the model—a higher score signifies a better fit to the data. Finally, mean absolute error (MAE) computes the average absolute difference between predicted and true values, offering robustness against outliers. In our creatinine regression experiments, we optimize our models by minimizing MSE, ensuring the models provide accurate predictions while being sensitive to potential outliers in the dataset.

#### *k*-fold cross-validation

Owing to our small dataset, we use *k*-fold cross-validation, a method that involves splitting the data into *k* subsets, or folds, for iterative model training and evaluation. *k*-fold cross-validation ensures better data utilization, and reduces overfitting risks associated with small datasets by using each sample for training and validation across different iterations. This approach enhances the reliability of performance estimation. Additionally, *k*-fold cross-validation fosters robustness in performance evaluation, overcoming sample-dependency issues present in single train/test splits. Averaging performance metrics from the *k* folds provides a more stable probabilistic performance assessment. Furthermore, *k*-fold cross-validation aids in model selection and hyperparameter tuning when working with limited data, enabling fair comparisons and effective optimization.

### Model training

**Table 2:** Hyperparameter values and tuning ranges within our experiments.

	Hyperparameter	Range
ANN	Number of hidden layers	1
	Hidden layer activation	ReLU
	Output layer activation (class.)	Sigmoid
	Output layer activation (reg.)	None
	Hidden layer neurons	4–64
	Dropout	0–0.5
	Learning rate	$10^{-4}$–$10^{-2}$
	Early stopping patience	3
RF	Number of trees	100–2000
	Maximum tree depth	10–200
	Minimum split samples	$\{ 2, 5, 10 \}$
	Minimum leaf samples	$\{ 1, 2, 4 \}$
	Maximum features	{sqrt, $\hbox {log}_2$, all}
	Bootstrapping	{T, F}

**Fig. 1:**
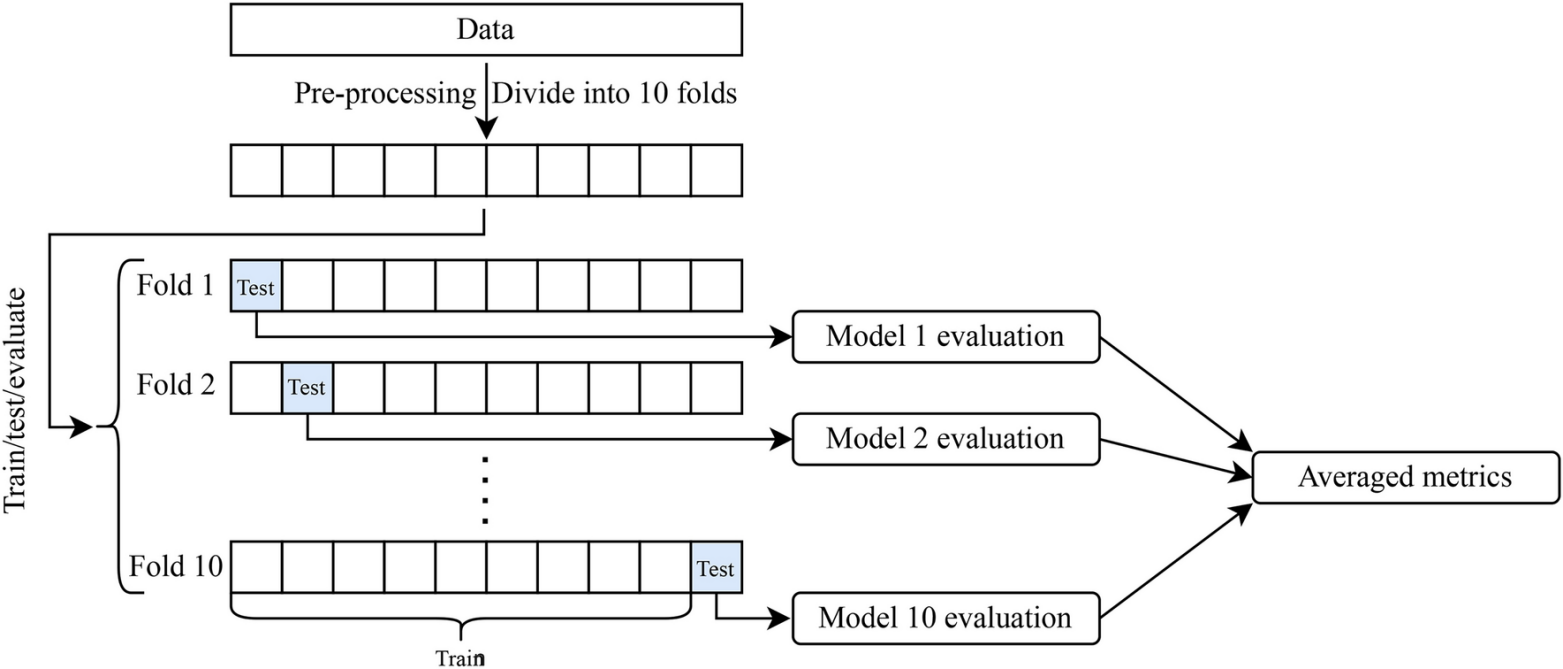
Block diagram of workflow. Starting with raw data, the data is imputed, one hot encoded, standardized, and divided into 10 folds. We then extract the at-home, monitoring, or laboratory features. Each train set is used to train a model, each trained model is then evaluated using its corresponding test set, producing individual model evaluations. The metrics from each model evaluation are averaged and the standard deviation is computed to produce the final result. We repeat this process for the three feature sets and two machine learning algorithms.

We first randomly split our data into 10 folds to use *k*-fold cross-validation with $k = 10$. In this way, we have a 90/10 train/test split for each fold. We then reserve 20% of the training data for validation. We implement our ANN models using Keras^21^, while we use scikit-learn^22^ for RF, both in Python. Within each fold, we tune the hyperparameters for the model. We show in Table 2 the values and ranges within the hyperparameter tuning. We use 50 trials in a random search using the Keras tuner^23^ and GridSearchCV within scikit-learn for ANN and RF, respectively. For RF the 50 trials are distributed among 5 inner folds. We then choose the best set of hyperparameters and re-train the model, and then evaluate the model on the test data. We repeat this process for both ANN and RF on each of the three features sets. We summarize our workflow in the block diagram shown in Fig. 1.

## Results

**Table 3:** CKD binary classification metric results presented as means of the 10 folds plus–minus the standard deviation.

	Features	Entropy	Accuracy	TPR	TNR	FPR	FNR
ANN	At-home	$0.349 \pm 0.116$	$82.9 \pm 9.93$	$92.0 \pm 7.00$	$67.9 \pm 34.8$	$32.1 \pm 34.8$	$8.03 \pm 7.00$
	Monitoring	$0.0800 \pm 0.0401$	$98.7 \pm 2.12$	$98.8 \pm 2.63$	$98.5 \pm 2.95$	$1.46 \pm 2.95$	$1.22 \pm 2.63$
	Laboratory	$0.0404 \pm 0.0541$	$99.2 \pm 1.69$	$98.8 \pm 2.50$	$100.0 \pm 0.00$	$0.00 \pm 0.00$	$1.19 \pm 2.50$
RF	At-home	$0.215 \pm 0.0680$	$92.5 \pm 4.08$	$90.0 \pm 6.63$	$95.8 \pm 5.50$	$4.23 \pm 5.50$	$9.96 \pm 6.63$
	Monitoring	$0.0714 \pm 0.0315$	$98.7 \pm 1.77$	$99.0 \pm 1.97$	$98.0 \pm 3.08$	$1.96 \pm 3.08$	$0.972 \pm 1.97$
	Laboratory	$0.0394 \pm 0.0199$	$99.5 \pm 1.05$	$99.6 \pm 1.25$	$99.2 \pm 2.50$	$0.833 \pm 2.50$	$0.417 \pm 1.25$

**Fig. 2:**
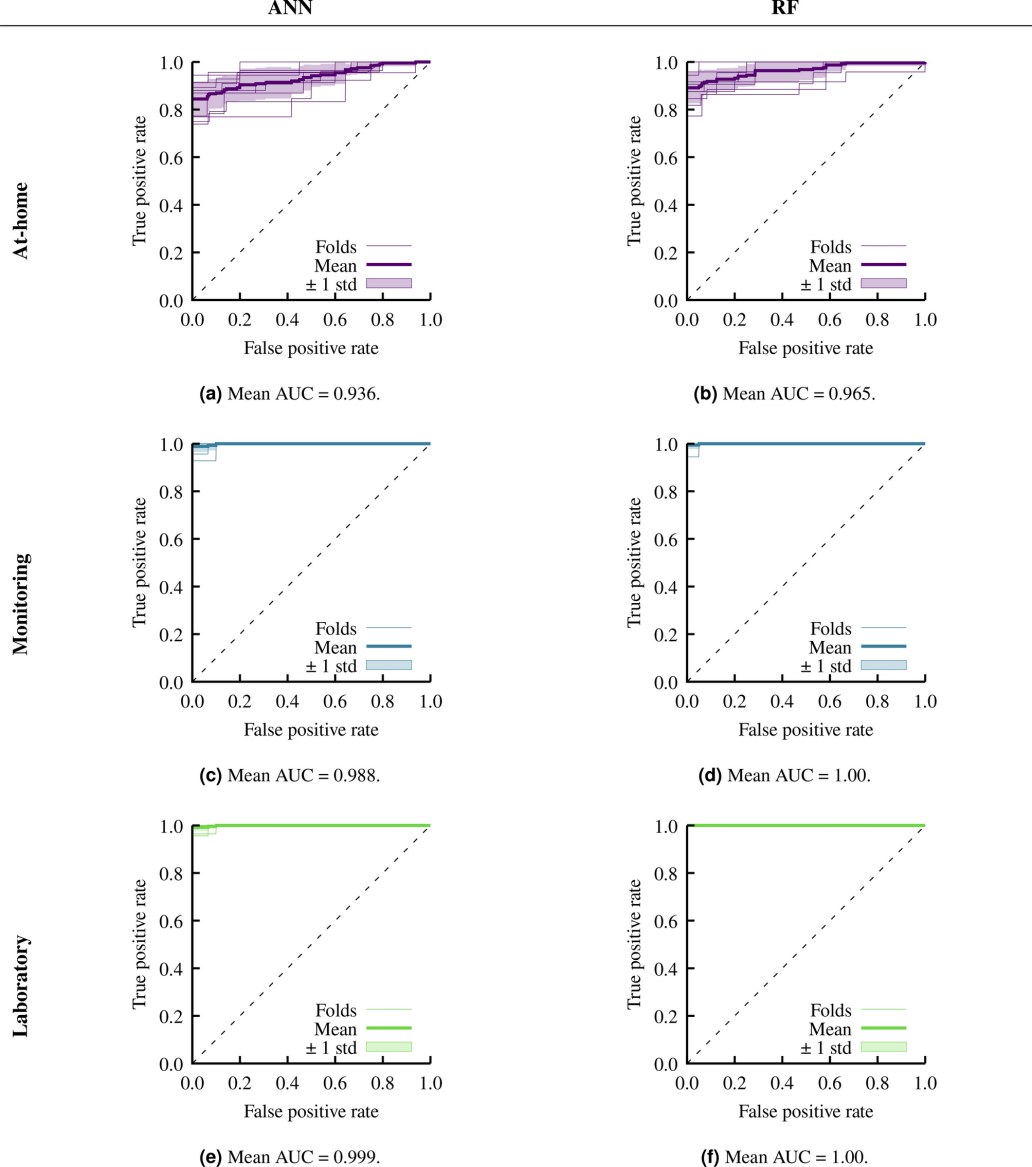
CKD binary classification ROC curves with AUC values.

**Fig. 3:**
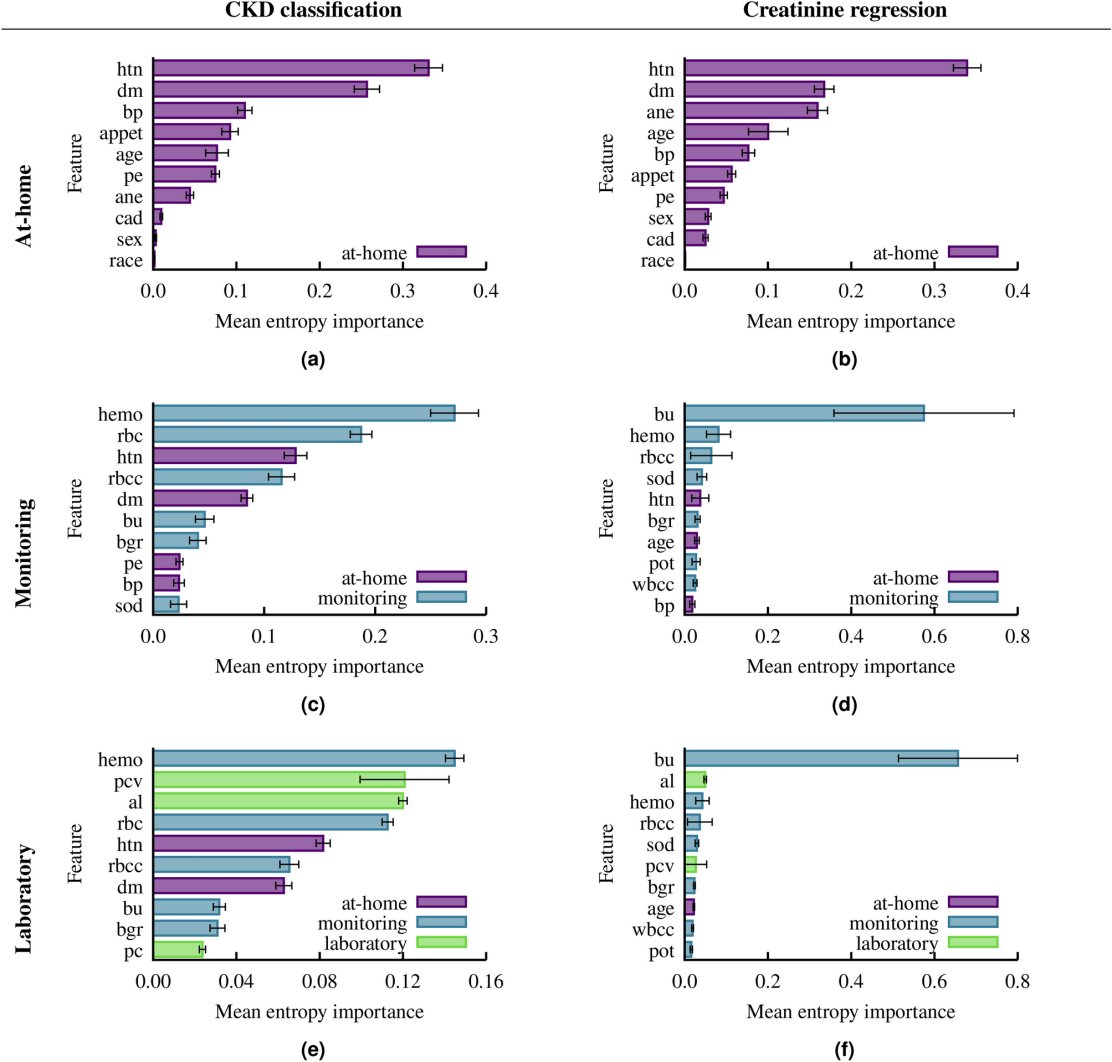
Mean entropy importance for the six experiments extracted from RF. The error bars denote the standard deviation.

After pre-processing the data, we are left with 383 patients with 54 features each (owing to the one hot encoding), and a split of 238/145 of CKD and not CKD. Of the 54 features, 27 and 18 make up the monitoring and at-home feature set, respectively. Using both ANN and RF we conduct both machine learning tasks on each feature set. We compute the metrics described in the previous section on the test data of each of the 10 folds, and compute the mean and standard deviation across the folds. We show our results using both ANN and RF to classify CKD in Table 3, and present creatinine prediction results in Table 4. Furthermore, in Fig. 2 we show the ROC curves and AUC values, and in Fig. 3 we show the feature importance extracted from the RF models.

### CKD classification

We show the results of our 10-fold cross-validation CKD classification in Table 3. We observe a marked difference in CKD classification metrics between the two machine learning methods with at-home features. Using at-home features, ANN achieves an average of 82.9%, 92.0%, and 67.9% for accuracy, TPR, and TNR, respectively, over the 10 folds. On the other hand, RF recovers a higher accuracy of 92.5%, a comparable 90.0% TPR, and a significantly higher 95.8% TNR. We note that the TPR for the at-home features is higher with ANN than RF, however, the higher accuracy and TNR of RF over ANN makes RF the better algorithm for at-home CKD classification. Accuracy of both ANN and RF CKD classification for both monitoring and laboratory features is over 98%. Furthermore, the TPR and TNR for both monitoring and laboratory features is also nearly perfect with rates exceeding 98%. There is little separation in the results for these two feature sets and the two methods. RF and ANN both perform worse with at-home features than with monitoring or laboratory features, but with the at-home feature set RF performs considerably better than ANN.

In Fig. 2 we show the ROC curves from the six classification tasks. We find similar performance between ANN and RF for all three feature sets. With the at-home features RF has a slightly higher AUC of 0.965 compared to 0.936 with ANN. With monitoring or laboratory features RF has an AUC of 1.00, while ANN is 0.998 and 0.999 for monitoring and laboratory features, respectively.

**Table 4:** Creatinine regression metric results presented as means of the 10 folds plus–minus the standard deviation.

	Features	MSE	R2	MAE
ANN	At-home	$0.614 \pm 0.170$	$0.284 \pm 0.190$	$0.585 \pm 0.0845$
	Monitoring	$0.295 \pm 0.202$	$0.674 \pm 0.182$	$0.387 \pm 0.0781$
	Laboratory	$0.300 \pm 0.0847$	$0.652 \pm 0.0773$	$0.416 \pm 0.0375$
RF	At-home	$0.583 \pm 0.134$	$0.381 \pm 0.203$	$0.551 \pm 0.0351$
	Monitoring	$0.319 \pm 0.133$	$0.682 \pm 0.0882$	$0.391 \pm 0.0456$
	Laboratory	$0.292 \pm 0.143$	$0.707 \pm 0.106$	$0.366 \pm 0.0524$

### Creatinine regression

We now turn our attention to the task of creatinine regression; we show our results in Table 4. The ANN and RF results are similar to each other. The only noticeable difference is in the R2 score for the at-home features. Here, RF performs better, however, with an average R2 score of 0.381, creatinine levels are not well predicted. In the remaining results, the method that performs better is evenly split between ANN and RF. As we saw with the classification, we again find there are only negligible differences between the monitoring and laboratory feature metrics. Once again, we find the at-home metrics are poorer than the monitoring or laboratory results. The MSE of the at-home regression is roughly double the MSEs of the monitoring and laboratory feature sets. One interesting result is that for ANN all three metrics were better for the monitoring features than with the full laboratory features.

### Random forest feature importance

Random forest allows us to estimate the importance of individual features from their frequency within the decision trees. In Fig. 3 we show the top 10 most important features (by mean entropy importance) for the six RF experiments. In the CKD classification problem for the monitoring features (Fig. 3c) and laboratory features (Fig. 3e) we find hemoglobin (hemo) is the most important feature in both groups. Hemoglobin has a mean entropy importance of 0.271 and 0.145, for the monitoring and laboratory features, respectively. Out of the top 10 most important classification laboratory features only three features are exclusive to the laboratory group. The overlap with the important monitoring features may help explain the small differences in metrics between the monitoring and laboratory feature sets as we saw in Table 3. Furthermore, the normal/abnormal red blood cell feature (rbc) is the second most important feature in the monitoring set with a mean entropy importance of 0.187, as well as the fourth most important feature in the laboratory set (0.113 mean entropy importance). We note in the laboratory features the red blood cell feature is only marginally less important than the packed cell volume (pcv), which has a mean entropy importance of 0.121. The top two features in the at-home set, hypertension (htn) and diabetes mellitus (dm), also appear in the top five features in the monitoring set, and are the fifth and seventh most important features in the full laboratory set. Both hypertension (htn) and diabetes mellitus (dm) play an important role in the 92.5% accuracy achieved with RF classification.

Turning to creatinine prediction, we again find hypertension (htn) and diabetes mellitus (dm) are the two most important features in the at-home set (Fig. 3b). However, anemia (ane) plays a more substantial role than in the classification task. In classification, blood urea (bu) was among the top 10 of both the monitoring and laboratory features. In the creatinine prediction, however, blood urea (bu) is by far the most important feature in both sets. Moreover, hemoglobin (hemo), red blood cell count (rbcc), and sodium (sod) are each in the top five of important features in the monitoring and laboratory sets. Hypertension (htn) drops to fifth most important in the monitoring features, and is not within the top 10 for laboratory features.

## Discussion

We separated the features into the three sets, as outlined in Table 1, based on the tools required for measurement. At-home features are known by a patient or are easily measurable, like age or blood pressure. Monitoring features are obtainable from regular check-ups, such as red blood cell count, and laboratory features are from blood and urine tests targeted towards CKD, such as urine albumin.

We have classified CKD and predicted creatinine levels on the three groups of features. In the classification case, we classified whether a person has CKD or not, disregarding stages. A patient’s GFR, and thus stage, can be estimated using the predicted creatinine level as an input into the CKD-EPI equation^8^. We carried out our experiments using both ANN and RF. Using the monitoring and laboratory feature sets, both ANN and RF had near perfect classification accuracy, and RF performed better than ANN using the at-home features. This is further highlighted by the ROC curves and AUCs obtained (Fig. 2). With monitoring and laboratory feature sets, ANN and RF both had AUCs of essentially 1. With the at-home feature set RF performed slightly better than ANN with a higher AUC, and a better ROC with a lower variance. Similarly, for creatinine regression both ANN and RF have comparable results using the monitoring and laboratory features, and RF performed better using at-home features. We observed some distinct performance differences between ANN and RF, particularly with at-home features. ANN achieved an average accuracy of 82.9%, a TPR of 92.0%, and a TNR of 67.9%, while RF performs better with a 92.5% accuracy, 90.0% TPR, and notably higher 95.8% TNR, making RF more suitable for at-home CKD classification due to its reduced false negative rate. For both monitoring and laboratory features, however, ANN and RF perform comparably, with accuracy, TPR, and TNR values all over 98%, showing high effectiveness with more comprehensive data. The ROC curves align with these results, with RF slightly outperforming ANN for at-home features (AUC of 0.965 vs. 0.936), while both methods show near-perfect AUC values with monitoring and laboratory features. For creatinine regression, RF achieves a higher R2 with at-home features (0.381), though this remains suboptimal, indicating limited predictive power without detailed clinical data. Overall, RF demonstrates a modest advantage with at-home features, while both models perform equally well when more extensive data is available. The advantage of RF over ANN when using at-home features is somewhat expected, as a majority of these features are categorical, and RF naturally handles categorical data more effectively than ANN due to the structure of decision trees. We expect the performance of ANN to improve with a more complex architecture, but this would require much more additional data.

Exploiting the nature of RF algorithms, we extracted the most important features in the classification and regression (Fig. 3). Hypertension and diabetes mellitus were the two most important features of the at-home set for both classification and regression as measured by the mean entropy importance. We had less agreement of the most impactful features between the classification and regression on the monitoring and laboratory feature sets. For monitoring features, blood urea and sodium are highly important for regression, but are less important for CKD classification. Red blood cell ranks second in classification, but does not appear in the top 10 for regression. Similarly, diabetes mellitus is among the top five in classification, but is much less significant for regression; hypertension remains important for both tasks. In the laboratory feature set, blood urea emerges as crucial for regression, while, again, red blood cell ranks fourth in classification, but is absent from the top 10 for regression. Hypertension is fifth in classification, but does not appear in the top 10 for regression. Hemoglobin was the most important feature for both monitoring and laboratory sets in the classification task, and both red blood cell count and hypertension were in the top five for both sets. When predicting creatinine, blood urea was by far the most important feature within the monitoring and laboratory sets, with hemoglobin, red blood cell count, and sodium being in the top five for both sets.

In our study, we leverage a dataset that has been examined in prior works^18,24–26^. The common thread among these studies, like one of our own, is the focus on CKD classification using all available features. Across these studies and our own (with monitoring or laboratory features), we consistently observe near-perfect accuracy in CKD classification, highlighting the robustness of machine learning methods. Presently, CKD diagnoses require laboratory measurements at least three months apart^6^, hence, machine learning could reduce wait time for a diagnosis and treatment plan.

Both Khalid et al.^24^ and Almansour et al.^25^ explore subsets of this dataset by either using only numerical features or by examining the performance with a reduced number of features, respectively. Specifically, Khalid et al.^24^ applied a 10-fold cross-validation approach using 14 features, including serum creatinine, and tested models such as gradient boosting, Gaussian Naive Bayes, decision tree, RF, and a hybrid model. They only report accuracy, and achieved an accuracy of 98% for RF and 100% for their hybrid model. Similarly, Almansour et al.^25^ employed support vector machine and ANN with 10-fold cross-validation, focusing on “best” feature subsets. They reported accuracies of 97.75% on the full feature set for both models and, notably higher accuracies of 98.5% and 98% using the best 12 features for support vector machine and ANN, respectively. Our results match or surpass these findings, as we achieve higher accuracy with both ANN and RF using the monitoring (98.7% for both) or laboratory (99.2% and 99.5%, respectively) feature sets. Additionally, our study contributes a novel perspective by categorizing features into at-home, monitoring, and laboratory subsets. Qin et al.^18^ tested multiple models, including logistic regression, RF, support vector machine, *k*-nearest neighbours, Naive Bayes, ANN, and an integrated model. They achieved over 99% accuracy, TPR, and TNR for logistic regression, RF, and the integrated model. Qin et al.^18^ identified key features such as specific gravity, hemoglobin, serum creatinine, albumin, packed cell volume, red blood cell count, hypertension, and diabetes mellitus as significant for CKD classification. Our findings align closely with theirs, underscoring the value of these attributes in CKD diagnosis. Pal^26^ employed support vector machine, RF, and ANN for binary classification using categorical, non-categorical, and combined feature sets, including serum creatinine. They reported accuracies of 88%, 92%, and 80% for support vector machine, RF, and ANN, respectively, along with TPRs of 61%, 55%, and 55% and AUC values of 0.77, 0.76, and 0.70, respectively. In contrast, our study achieves near-perfect results using the laboratory or monitoring feature sets (while excluding serum creatinine), with both ANN and RF models achieving a mean accuracy exceeding 99% and AUC values of 0.999 and 1.00, respectively. Unlike previous studies that focus on subsets of “best” features, we emphasize grouping data that is typically collected together, which provides a practical framework for CKD prediction based on context-specific data availability. Our approach enhances the interpretability of our models and provides insights into the relevance of features for different aspects of CKD prediction.

We employed a similar approach to Wang et al.^14^ by predicting creatinine levels directly. Their ensemble method achieved an R2 score of 0.5590 using a different dataset, and they emphasize the significance of hemoglobin. In our findings, hemoglobin was in the top three features for both monitoring and laboratory features for creatinine prediction. We note the data used in Ref.^14^ did not contain blood urea, which we found to be the most significant predictor.

A comprehensive overview of machine learning techniques for CKD classification can be found in Ref.^11^, where a wide range of methods are assessed. Notably, the best-performing models in their tabulation achieve accuracies of 98% or higher. Sanmarchi et al.^12^ conducted a review encompassing 68 relevant articles on CKD prediction, diagnosis, and treatment using a wide range of machine learning methods. Their findings emphasize the importance of attributes such as blood pressure, hemoglobin, sodium, albumin, pus cell, red blood cell count, and diabetes mellitus for CKD prognosis and diagnosis. These highlighted features align with our own feature importance observations, accentuating their relevance in the context of CKD assessment.

In summary, our study builds upon a dataset examined in previous research and offers a unique perspective by categorizing features into context dependent subsets. Our findings, including the importance of specific attributes and the success of machine learning methods in CKD classification, corroborate and extend upon existing literature, contributing to a better understanding of CKD detection and prediction.

Our study has some limitations however, that warrant consideration. Firstly, while the CKD and not CKD labels in the dataset were assigned by nephrologists using patient history, symptoms, and blood and urine tests, it is not clear precisely what criteria were used to determine the labels. Moreover, the absence of stage-specific information for patients with CKD in our dataset poses a challenge. Additionally, our models’ ability to detect early-stage CKD may be limited, as we primarily focused on classifying CKD in general, not the stage. Having labelled data with explicit CKD stages would enable a more nuanced analysis and classification of disease progression. Presently, our creatinine regression results provide a proxy for stage, but this transition from creatinine to stage introduces additional uncertainty, given the equations involved are empirical in nature. These limitations highlight the need for more comprehensive and stage-specific datasets to further improve the accuracy and clinical relevance of CKD detection and classification models. We also acknowledge the necessity for larger and more comprehensive datasets to enhance the precision and applicability of our models. Additionally, incorporating a more diverse population is crucial to ensure a model’s robustness across various demographics and regions. These improvements would lead to more accurate and generalizable predictions in real-world clinical settings.

## Conclusions

Our study represents a step toward leveraging machine learning for the early detection and classification of CKD, addressing a pressing concern in clinical medicine. By examining different feature subsets of at-home, monitoring, and laboratory, we offer insights into the potential use of such models in various clinical settings. Our findings, which align with previous research, stress the importance of specific features such as blood urea, hemoglobin, blood pressure, and diabetes mellitus in CKD detection and classification. Looking ahead, the impact of this work extends to the development of more robust and accurate CKD screening tools, potentially facilitating earlier interventions and improved patient outcomes. However, we recognize the need for larger and more comprehensive datasets, including detailed CKD stage information, to enhance the precision of our models. Additional, high-quality, diverse data will help improve the accuracy of ANN CKD classification accuracy. Furthermore, training a dual-task ANN to simultaneously classify CKD and predict creatinine may improve the performance of both tasks. With continued research and access to richer clinical data the integration of machine learning techniques into routine CKD diagnosis and prognosis holds the promise of assisting the field of nephrology. Machine learning could improve the lives of many individuals affected by this pervasive health condition.

## Supplementary Information

Supplementary Information.

## Data Availability

The datasets used during the study are available from the corresponding author upon request.
