# Supplementary material for: Machine learning for classifying chronic kidney disease and predicting creatinine levels using at-home measurements
Source: Sci Rep. 2025 Feb 5;15:4364. doi: 10.1038/s41598-025-88631-y (PMC11799517; doi:10.1038/s41598-025-88631-y)
Supplement: Supplementary file 1 — Supplementary Information. [file 41598_2025_88631_MOESM1_ESM.pdf]

Machine learning for classifying chronic kidney disease and predicting creatinine levels using at-home measurements

Supplementary information

Brady Metherall [1,*], **Anna K. Berryman** [1], **and Georgia S. Brennan** [1]

[1]Mathematical Institute, University of Oxford, Radcliffe Observatory Quarter, Andrew Wiles Building, Woodstock Rd, Oxford, OX2 6GG, United Kingdom
[*]metherall@maths.ox.ac.uk

Descriptive statistics of dataset

See Table S1 for descriptive statistics of the numerical features and Table S2 for descriptive statistics of the nominal features.

Table S1. Descriptive statistics of numerical features.

	age	bp	bgr	bu	sc	sod	pot	hemo	pcv	wbcc	rbcc
count	391	388	356	381	383	313	312	348	329	294	269
mean	51.48	76.47	148.04	57.43	3.07	137.53	4.63	12.53	38.88	8406.12	4.71
std	17.17	13.68	79.28	50.50	5.74	10.41	3.19	2.91	8.99	2944.47	1.02
min	2.00	50.00	22.00	1.50	0.40	4.50	2.50	3.10	9.00	2200.00	2.10
25%	42.00	70.00	99.00	27.00	0.90	135.00	3.80	10.30	32.00	6500.00	3.90
50%	55.00	80.00	121.00	42.00	1.30	138.00	4.40	12.65	40.00	8000.00	4.80
75%	64.50	80.00	163.00	66.00	2.80	142.00	4.90	15.00	45.00	9800.00	5.40
max	90.00	180.00	490.00	391.00	76.00	163.00	47.00	17.80	54.00	26400.00	8.00

Table S2. Descriptive statistics of nominal features.

	sg	al	su	rbc	pc	pcc	ba	htn	dm	cad	appet	pe	ane	race	sex	class
count	400	400	400	248	335	396	396	398	398	398	399	399	399	400	399	400
unique	6	7	7	2	2	2	2	2	2	2	2	2	2	2	2	2
top	1.02	0.0	0.0	norm.	norm.	notpres.	notpres.	no	no	no	good	no	no	notAA	m	ckd
freq	106	199	290	201	259	354	374	251	261	364	317	323	339	393	201	250
